# Maternal pre-pregnancy BMI associates with neonate local and distal functional connectivity of the left superior frontal gyrus

**DOI:** 10.1038/s41598-021-98574-9

**Published:** 2021-09-28

**Authors:** Olli Rajasilta, Suvi Häkkinen, Malin Björnsdotter, Noora M. Scheinin, Satu J. Lehtola, Jani Saunavaara, Riitta Parkkola, Tuire Lähdesmäki, Linnea Karlsson, Hasse Karlsson, Jetro J. Tuulari

**Affiliations:** 1 FinnBrain Birth Cohort Study, Turku Brain and Mind Center, Institute of Clinical Medicine, University of Turku, Lemminkäisenkatu 2, 20520 Turku, Finland; 2 Department of Psychiatry, University of Turku and Turku University Hospital, Turku, Finland; 3 Department of Psychiatry, University of Oxford, UK (Sigrid Juselius Fellowship), Oxford, UK; 4 Department of Medical Physics, Turku University Hospital, Turku, Finland; 5 Department of Radiology, University of Turku and Turku University Hospital, Turku, Finland; 6 Department of Pediatric Neurology, Turku University Hospital and University of Turku, Turku, Finland; 7 Turku Collegium for Science, Medicine and Technology, University of Turku, Turku, Finland; 8 Department of Psychiatry for Affective Disorders, Sahlgrenska University Hospital, Gothenburg, Sweden; 9 Department of Clinical Neuroscience, Karolinska Institutet, Stockholm, Sweden; 10 Center for Population Health Research, University of Turku and Turku University Hospital, Turku, Finland; 11 Department of Neurology, University of California, San Francisco, San Francisco, CA USA

**Keywords:** Development of the nervous system, Obesity

## Abstract

Maternal obesity/overweight during pregnancy has reached epidemic proportions and has been linked with adverse outcomes for the offspring, including cognitive impairment and increased risk for neuropsychiatric disorders. Prior neuroimaging investigations have reported widespread aberrant functional connectivity and white matter tract abnormalities in neonates born to obese mothers. Here we explored whether maternal pre-pregnancy adiposity is associated with alterations in local neuronal synchrony and distal connectivity in the neonate brain. 21 healthy mother-neonate dyads from uncomplicated pregnancies were included in this study (age at scanning 26.14 ± 6.28 days, 12 male). The neonates were scanned with a 6-min resting-state functional magnetic resonance imaging (rs-fMRI) during natural sleep. Regional homogeneity (ReHo) maps were computed from obtained rs-fMRI data. Multiple regression analysis was performed to assess the association of pre-pregnancy maternal body-mass-index (BMI) and ReHo. Seed-based connectivity analysis with multiple regression was subsequently performed with seed-ROI derived from ReHo analysis. Maternal adiposity measured by pre-pregnancy BMI was positively associated with neonate ReHo values within the left superior frontal gyrus (SFG) (FWE-corrected *p* < 0.005). Additionally, we found both positive and negative associations (*p* < 0.05, FWE-corrected) for maternal pre-pregnancy BMI and seed-based connectivity between left SFG and prefrontal, amygdalae, basal ganglia and insular regions. Our results imply that maternal pre-pregnancy BMI associates with local and distal functional connectivity within the neonate left superior frontal gyrus. These findings add to the evidence that increased maternal pre-pregnancy BMI has a programming influence on the developing neonate brain functional networks.

## Introduction

Maternal obesity (BMI ≥ 30 kg/m^2^) and overweight (BMI ≥ 25–30 kg/m^2^) during pregnancy have become prevalent worldwide within the last few decades^1^. While the risks of obesity and overweight pregnancies have been extensively studied from obstetric point of view, and is identified as a risk factor for delivery and congenital structural abnormalities^1^, less is known about its association to child neurodevelopment. Studies focusing on neurobehavioral and neurodevelopmental aspects have linked maternal obesity and overweight during pregnancy to impaired offspring cognitive development^2,3^, emotional/behavioural problems and consecutive increased risk in obtaining a diagnosis for neuropsychiatric disorders, including anxiety and depressive disorders, autism spectrum disorder, attention deficit hyperactivity disorder and even psychotic disorders^2,3^.

Obesity and overweight are related to complex alterations in metabolism, e.g. insulin resistance, increased circulating levels of lipids, dysfunction of adipose tissue and skeletal muscle, hepatic and pancreatic tissue as well as low grade oxidative stress and inflammation^4–6^. During pregnancy, the adverse inflammatory processes associated with obesity and overweight may cause placental dysfunction^7^, likely increasing fetal vulnerability to endo- and exogenous exposures through altered placental vascular permeability. Obesity and overweight are also accompanied by humoral dysregulation with increased levels of estrogen and adipokines, e.g. leptin^8^, which may further contribute to placental dysfunction^6^. These metabolic, humoral and inflammatory alterations coupled with possible placental dysfunction are highly plausible factors to exert a programming effect on the developing fetal brain. Animal model investigations into maternal obesity and offspring brain development have provided some insight on the mechanisms, including dysregulation within serotonergic and dopaminergic systems^9,10^, altered hypothalamic–pituitary–adrenal axis (HPA-axis) responses^11^, fetal neuronal damage^12^ and changes in offspring brain gene expression patterns^12^. Indeed, these animal models have further suggested that circulating immune mediators in obesity have programming effects on offspring microglia, causing alterations in activity and different baseline for reactivation^13,14^. As microglia are important modulators of neurogenesis, synaptogenesis, neurite formation, axonal fasciculation and synaptic pruning, divergent microglia activity may very well induce changes in macro-scale functional networks^13,14^. Finally, maternal breast milk of obese mothers contain a fatty acid composition inclined towards pro-inflammation at the expense of fatty acids important for neurodevelopment^15^.

Recent advances in brain imaging techniques, such as diffusion tensor imaging (DTI) and resting-state functional magnetic resonance imaging (rs-fMRI), have provided the opportunity to probe gestational effects of various states and factors on human fetal and neonate brain development^16^, including maternal obesity and overweight. In adults, resting-state networks (RSNs) have been shown to remain stable over time with little variability over imaging sessions, revealing the distributed intrinsic functional organization of the brain where long-range connections dominate^17^. During the first year of life, there is a formidable gradual shift from local, intra-hemispheric network connectivity seen already in utero and in the neonatal stage to more distributed network connectivity in older children and adults^18–20^. These macro-scale network changes are described with prominent alterations to functional hub localization, proliferation of connector hubs and progression of functional segregation of networks, likely indicating more efficient information processing within and between networks over the first postnatal years in normal development^19,21,22^. Further, alterations in network topology temporally coincide with increased white matter myelination^23^ and synaptic pruning^24^. The delicate developmental and reconfiguration processes in brain functional networks during gestation and the first years, respectively, present a time window in brain development, that has been shown to be particularly vulnerable for disruption by endogenous and exogenous factors^16^. Prior human MRI neonate studies focusing on obese and overweight pregnancies have revealed that maternal adiposity is associated with widespread alterations in the anterior brain white matter tract integrity^25^ and in functional networks^26,27^ with emphasis on sensory cue and reward processing, cognitive and motor control in the neonate brain^28^.

Regional homogeneity (ReHo) is an efficient, reliable and widely used index of local fMRI connectivity^29,30^. Based on the assumption that hemodynamic characteristics of every voxel in a functional cluster should be similar to the neighbour voxels, ReHo is commonly interpreted as an index of ongoing brain activity^29^. ReHo measured at rest is altered in many canonical RSNs^31^ and those that involve the social brain^32^ in neonates with family history of autism and adolescents with autism^33^. In adults, ReHo shows promising sensitivity to functional changes in schizophrenia^34^, cognitive impairment^35^ and even pre-symptomatic stages of genetic dementia^36^. Recent findings have revealed infant brain hemodynamics to show large response variability to experimental design and presented stimulus, further complicating comparison between study populations regarding age. ReHo, however, can circumvent this problem by computing the local connectivity, e.g. positive or negative hemodynamic response, of neighbouring voxels using Kendall’s correlation coefficient. This alleviates the comparison problem between age groups, as the direction of hemodynamic response becomes redundant. To the best of our knowledge, there have been no investigations into maternal adiposity induced alterations in local connectivity of the neonate brain. In the present study, we hypothesized that correlations between maternal pre-pregnancy BMI and ReHo may reveal functional abnormalities associated with altered neurodevelopment. Specifically, maternal obesity may be positively associated with ReHo values as a sign of delayed neurodevelopment, especially in the frontal areas^28,37–39^. For future reference we also provide the average ReHo maps of the neonate brain at 26.14 ± 6.28 days after birth in the supplementary materials. Further, we implemented seed-based connectivity analysis (SCA) to investigate whether alterations in ReHo are reflected in distal functional connectivity.

## Materials and methods

This study was conducted in accordance with the Declaration of Helsinki, and it was approved by the Ethics Committee of the Hospital District of Southwest Finland (15.03.2011) §95, ETMK: 31/180/2011. Informed written consents were obtained from parents before MRI scans were conducted.

### Participants

This study was performed as a part of FinnBrain Birth Cohort Study (www.finnbrain.fi)^40^. 28 dyads of full-term born healthy infants and mothers (Table 1) were randomly recruited from the cohort and participated to fMRI scans (performed during year 2015). Exclusion criteria for infants included complications of neurological involvement, less than 5 points in the 5 min Apgar, previously diagnosed central nervous system anomaly, gestational age at delivery less than 32 weeks and birth weight less than 1500 g. Seven dyads were excluded from the study due to excessive neonate motion during the MRI scanning session. All mothers reported having stopped ingesting alcohol and possible use of illicit substances after being informed of being pregnant, although three participants with minor exposure to alcohol or illicit substances (cannabis) during early gestation were included. The sample likely reflects the general Finnish population. None of the included mothers suffered from hypertension, hypercholesterolemia or any form of diabetes mellitus. All scans were carried out during natural sleep at the gestation corrected age of 26.14 ± 6.28 days. To facilitate natural sleep, infants were fed with (breast) milk prior to the scanning session.

**Table 1:** Sample demographics of included dyads (N = 21) comprising of neonates and mothers that participated in this study.

Variable	Whole sample (N = 21)	Boys (N = 12)	Girls (N = 9)
**M ± SD (range)**			
Age from birth (days)	26.95 ± 9.01 (11–53)	24.50 ± 7.67 (11–36)	30.22 ± 10.07 (23–53)
Age from term (days)	26.14 ± 6.28 (17–45)	23.17 ± 4.26 (17–30)	30.11 ± 6.23 (23–45)
Gestational age when born (weeks)	43.78 ± 0.91 (42.43–46.43)	43.39 ± 0.71 (42.43–44.43)	44.30 ± 0.93 (43.43–46.43)
Offspring birth weight (grams)	3524.76 ± 338.05 (3085–4395)	3562.50 ± 295.82 (3105–3980)	3474.33 ± 400.45 (3085–4395)
Offspring head circumference when born (cm)	35.29 ± 1.22 (33.0–37.5)	35.67 ± 1.21 (34.0–37.5)	34.78 ± 1.09 (33.0–37.0)
Maternal age (years)	28.95 ± 4.20 (19–37)	29.08 ± 4.78 (19.00–37.00)	28.78 ± 3.56 (24.00–36.00)
Maternal pre-pregnancy BMI (kg/m2)	25.57 ± 4.05 (20.03–34.42)	25.92 ± 4.49 (20.03–34.42)	25.10 ± 3.59 (21.05–33.06)
Apgar points at 1 min (MAD)	8.38 (1.15)	8.08 (1.22)	8.78 (0.25)
Apgar points at 5 min (MAD)	9.05 (0.46)	9.00 (0.49)	9.11 (0.40)
Maternal EPDS-score	4.7 ± 4.2 (0–17)	3.8 ± 2.4 (0–8)	5.9 ± 5.7 (1–17)
**Frequencies**			
Maternal pre-pregnancy BMI (kg/m^2^) (1 = < 25.00 / 2 = 25.00–29.99 / 3 = ≥ 30)	10/7/4	6/3/3	4/4/1
Maternal monthly income (€) (1 = < 500/2 = 501–1000/3 = 1001–1500/4 = 1501–2000/5 = 2001–2500/6 = 2501–3000/7 = 3001–3500/8 = 3501–4000/9 = > 4000)	2/1/2/11/4/1/0/0/0	1/0/2/5/4/0/0/0/0	1/1/0/6/0/1/0/0/0
Maternal education level (1 = High school graduate or lower; 2 = College degree; 3 = University degree)	5/7/9	2/3/7	3/4/2
Race/Ethnicity (Caucasian/other)	21/0	12/0	9/0
Maternal use of alcohol during pregnancy (yes/no)	3/18	2/10	1/8
Frequency of maternal use of alcohol during pregnancy (More than 1–2 times a month / 1–2 times a month / less frequently)	0/1/2	0/1/1	0/0/1
Maternal use of illicit substances during pregnancy (yes/no)	1/20	0/12	1/8
Frequency of maternal use of illicit substances during pregnancy (More than 1–2 times a month /1–2 times a month / less frequently)	0/0/1	0/0/0	0/0/1

Variable selection was based on previous recommendations^41^. Abbreviations: M = Mean; SD = Standard deviation; MAD = Mean absolute deviation; EPDS = Edinburgh postnatal depression scale 10-point questionnaire sum score filled out at 24th gestational week.

### Measures and procedures

Obstetric data were obtained from the Finnish Medical Birth Register of the National Institute for Health and Welfare and included age from birth and term, gestational age when born, Apgar points at 1 and 5 min, gestational weight, head circumference, maternal age in years, race/ethnicity, maternal pre-pregnancy BMI and exposure to alcohol and/or illicit substances. Education levels were trichotomized (low: high school or lower; middle: college degree; high: university degree). Maternal symptoms of depression were measured by Edinburgh postnatal depression symptom (EPDS) 10-point questionnaire, filled out by mothers during 24th gestational week. Variable selection was based on previous recommendations^41^. EPDS was chosen as a proxy for maternal psychological distress as prior reports have indicated that maternal depressive symptoms may reflect such distress that can affect offspring development^42^.

### Image acquisition

28 infants underwent an MRI brain scanning session, including a 6 min resting-state fMRI sequence. MRI scans were conducted on a Siemens Magnetom Verio 3T MRI scanner (Siemens Medical Solutions, Erlangen, Germany) equipped with a 12-element Head Matrix coil. Field-of-view (FOV) parameters were optimized for future replication by linear alignment to the anterior and posterior commissure line. The total duration of the scanning protocol was 60 min, comprising of five major sequences in the following order: (1) Axial PD-T2-weighted TSE (Turbo Spin Echo), (2) Sagittal T1-MPRAGE (Magnetization Prepared Rapid Acquisition Gradient Echo), (3) GRE field mapping, (4) DTI and (5) a 6-min rs-fMRI EPI (Echo-Planar Imaging) sequence. Fat saturation was applied and following acquisition parameters were used in rs-fMRI sequence: TR of 2500 ms, TE of 30 ms, FOV of 216 × 216 mm^2^, flip-angle (FA) of 80 degrees, GRAPPA acceleration of 2 and bandwidth of 1310 Hz/Px. Acquired rs-fMRI data consisted of 140 volumes with 42 slices and a voxel size of 3.0 × 3.0 × 3.0 mm^3^.

### Image preprocessing

#### ReHo

Data were slice timing corrected and motion corrected in FMRIB Software Library (FSL)^43^ v6.0 relative to a manually chosen reference volume, free of major artefacts. Motion outliers were estimated by ART (http://www.nitrc.org/projects/artifact_detect; Composite motion threshold (CMT) < 2 mm, DVARS < 9). As neonates commonly exhibit more movements in the scanner than older infants and adults, more stringent CMT cutoff values would have resulted in considerable increase in rejection rate of available data. At this initial step, rs-fMRI data of seven subjects were rejected from further analyses based on major artefacts (with most having ca. 4/6 min of data outliers), yielding an included sample size to 21. Anatomical masks for white matter and CSF were defined by the UNC neonate segmentation model^44^ and registered to functional data with affine transformation. Average signal in white matter average and CSF as well as 24 motion covariates^45^ were included as nuisance covariates. Thus, denoising consisted of nuisance regression followed by outlier rejection, detrending, and high-pass filtering (0.008 Hz).

The main outcome metric for functional organization of the neonate brain was ReHo, which is estimated in a data-driven manner and provides a voxel-wise, local connectivity measure across the whole brain^29,46^. ReHo is based on calculating the Kendall’s coefficient of concordance over a target voxel and neighboring voxels. ReHo was computed as implemented in DPABI (number of voxels in a cluster; N = 27) (http://rfmri.org/DPABI). For group analysis, ReHo maps were normalized to the UNC neonate template with 1.0 × 1.0 × 1.0 mm^3^ voxel dimensions. Finally, the data were smoothed with a Gaussian filter of 6 mm full width at half maximum (FWHM).

#### SCA

The SCA analyses were performed with FSL tools with the use of identical preprocessing and nuisance regression as for the ReHo analyses (see above). Seed region-of-interest (ROI) was defined by a 3 mm radius sphere generated in FSL’s^43^ FSLeyes, corresponding the location (left superior frontal gyrus; left SFG) of our ReHo result in UNC neonate template space. Seed-based connectivity maps were then generated using FSL v6.0 fMRI expert analysis tool (FEAT)^47^. Seed ROIs were warped from template to subject space before extracting time series information. To obtain subject-specific inverse transformation, we first applied FMRIB’s linear image registration tool (FLIRT)^48^ with 12 degrees-of-freedom, referencing a custom fMRI template obtained by averaging preprocessed fMRI data in UNC neonate template space. Subsequently, FMRIB’s nonlinear image registration tool (FNIRT) initialized by the affine matrix was used to estimate warps from subject to template space. These warping coefficients were then inverted by using FSL’s ‘invwarp’ command, and used to accurately transform the seed masks into the native space of each subject with the ‘applywarp’ command and nearest neighbor interpolation. Finally, average time series from seed ROIs were extracted using the ‘fslmeants’ command and a first-level FEAT analysis ran to assess the brain regions that have activity correlated to the mean left SFG ROI activity. The resulting z-score maps for each participant were then normalized to UNC template space and group-level statistical analyses conducted in SPM12, matching the ReHo analysis.

### Statistical analysis

All statistical analyses were performed with SPM12 (https://www.fil.ion.ucl.ac.uk/spm/software/spm12/) software with general linear models (GLM), SPM’s multiple regression design for ReHo and SCA maps. Maternal pre-pregnancy BMI was set as the main explanatory variable (EV), and gestation corrected age and neonate sex were set as primary independent variables (IV). The control for false positives is of paramount importance^49^. We set the a priori threshold for voxel-level statistical significance to *p* < 0.005 and FWE-corrected at the cluster level and verified that all results survive the non-parametric statistical testing (SnPM13; www.warwick.ac.uk/fac/sci/statistics/staff/acamedic-research/nichols/software/snpm). We also systematically explored whether the results survive a more stringent thresholding at *p* < 0.001 FWE-corrected at the cluster level. Images were inclusively masked after cluster correction with averaged UNC template GM mask to limit the statistics to the grey matter. For ReHo maps, we ran separate sensitivity analyses with identical design except for the added fourth regressor of no interest for the following: Apgar points at 1 and 5 min, neonate birth weight, maternal age in years and EPDS questionnaire score filled out by mothers at the 24th gestational week. Models with Apgar points at 1 and 5 min were performed with Statistical nonparametric mapping due to non-normal distribution of the Apgar data. To compute mean seed-based connectivity, we ran “one-sample T-test” for subject level SCA maps. To compare seed connectivity between groups, we performed “two-sample T-test” with maternal pre-pregnancy BMI cut-off of 25.0 kg/m^2^. There were 10 subjects within the overweight/obese group and 11 subjects in the control group. Subsequently, we performed multiple regression analysis with identical design as in the ReHo analyses. All models were replicated with SnPM13. We applied the a priori threshold for voxel-level statistical significance to *p* < 0.005 and FWE-corrected at the cluster level and included a more lenient thresholding at *p* < 0.05 and FWE-corrected for the SCA statistics only. Voxel-wise results were visualized with Mango software version 4.0.1 (www.ric.uthscsa.edu/mango).

Finally, to delineate whether motion estimates had effect on our SPM models, we performed a correlation analysis between three motion estimates and maternal pre-pregnancy BMI. We found no statistically significant correlation between motion estimates derived from MCFLIRT and maternal pre-pregnancy BMI (mean displacement r_s_ = − 0.232, *p* = 0.387; estimated rotations r_s_ = 0.003, *p* = 0.991; estimated translations r_s_ = − 0.205, *p* = 0.447).

## Results

### ReHo

**Figure 1:**
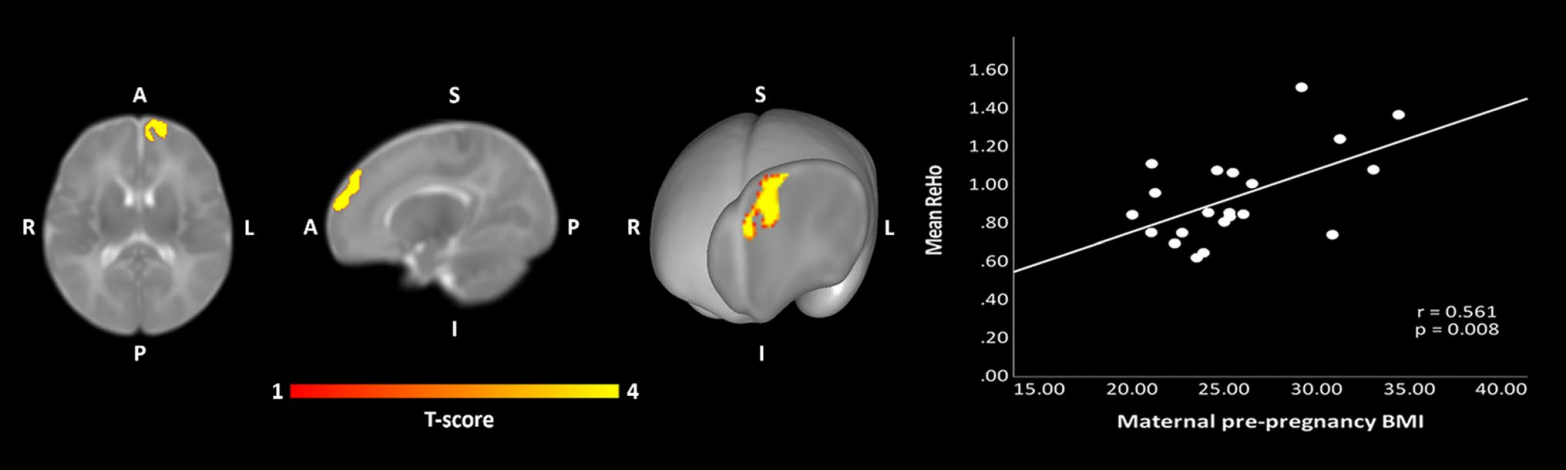
Regions where ReHo significantly correlated with maternal pre-pregnancy BMI (*p* < 0.005 threshold; *p* < 0.004 FWE-corrected) highlighted left SFG within the naturally sleeping neonate (N = 21). Color bar represents T-scores. Images are displayed in radiological convention on the UNC template in axial, sagittal and oblique slices. Oblique section was created with Mango surface builder. Abbreviations: ReHo = regional homogeneity; SFG = superior frontal gyrus; A = Anterior, P = Posterior, R = Right, L = Left, S = Superior, I = Inferior.

Multiple regression analysis for neonate brain ReHo maps and maternal pre-pregnancy BMI revealed a positive association [*p* < 0.005 (*p* < 0.002 FWE-correction, cluster size (kE) 869 voxels)] for left superior frontal gyrus (SFG); as identified from the UNC-neonate-atlas^44^. ReHo values were principally increased in the dorsal and medial aspects of the left SFG (Fig. 1). Cluster coordinates are displayed in Supplementary materials, Table 1. No negative associations were detected between maternal pre-pregnancy BMI and neonate ReHo maps.

In the performed sensitivity analyses (Supplementary materials, Tables 1, 2), Apgar points at 1 and 5 min after birth did not have any statistically significant effect on the ReHo-BMI correlation maps. Including maternal age as an additional IV to the original model reduced the original effect to statistical insignificance at *p* < 0.05 level (explained by the high correlation of r_S_ = 0.570 between maternal age and pre-pregnancy BMI). Maternal age had no independent statistically significant effects on neonate ReHo maps at *p* < 0.005 or lenient thresholds as investigated by a separate model with neonate age, sex and maternal age as covariates.

**Figure 2:**
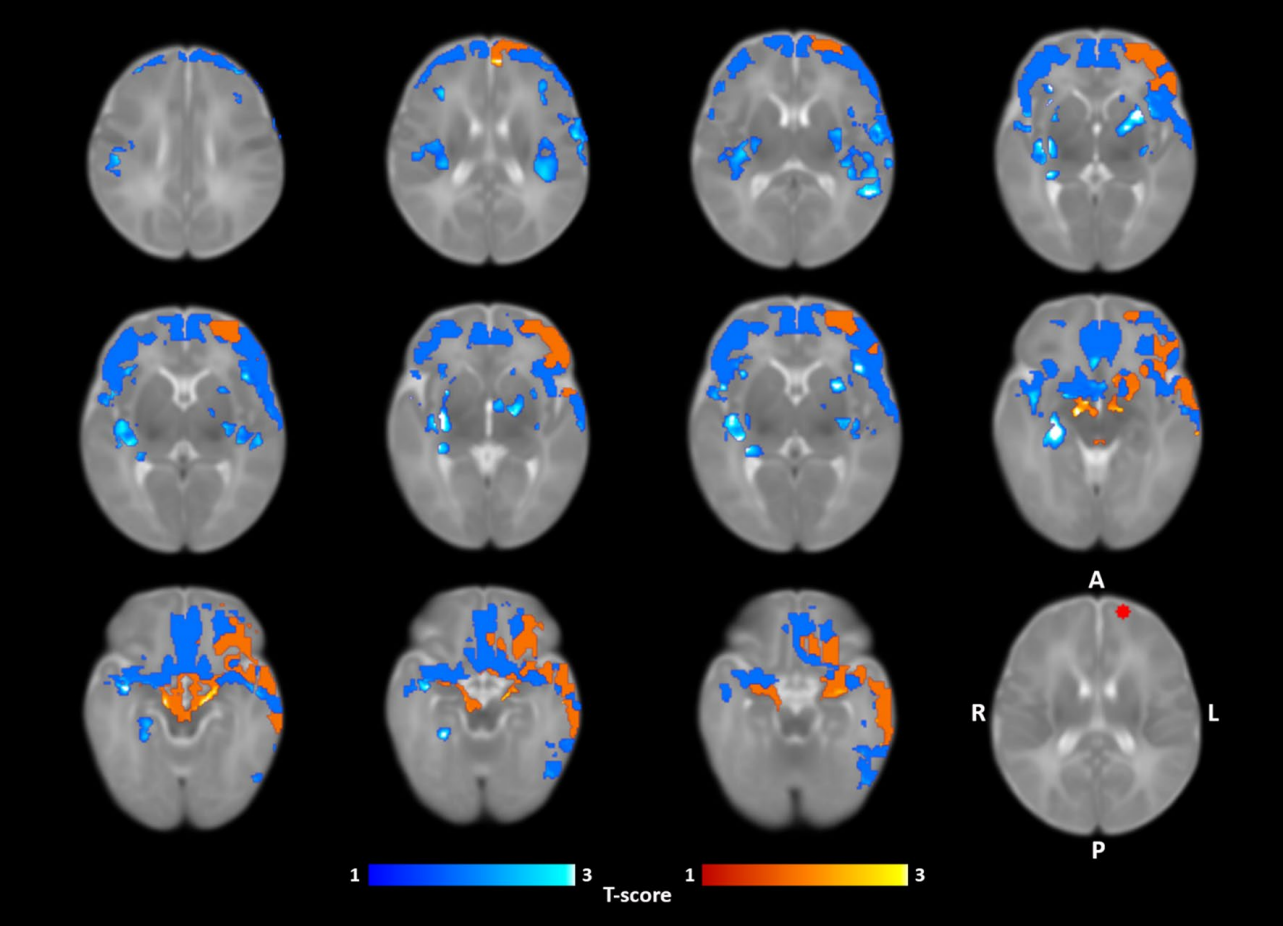
Regions where functional connectivity of the left SFG was positively (orange) and negatively (blue) correlated with maternal pre-pregnancy BMI (*p* < 0.05 threshold; *p* < 0.001 FWE-corrected) in the sleeping neonate (N = 21). Bottom right image displays the left SFG seed ROI in red color (coordinates on the UNC neonate template: X = 79, Y = 157, Z = 86). Color bars represent t-scores. Images are displayed in axial slices on the UNC neonate template. Abbreviations: SFG = Superior frontal gyrus, A = Anterior, P = Posterior, R = Right, L = Left.

IVs that had effects on the original model included offspring birth weight and EPDS sum score filled out at the middle of the 24th gestational week (Supplementary materials, Sect. 2, Figs. 2–5). Neither had independent statistically significant effects on neonate ReHo maps at *p* < 0.005 or lenient thresholds. Including infant birth weight as a fourth IV increased the statistical significance of maternal pre-pregnancy BMI effect on neonate ReHo-BMI maps (at *p* < 0.001 level; cluster size of 609 voxels and at *p* < 0.005 level with cluster size of 1437 voxels) with similar spatial distribution. Infant birth weight and maternal pre-pregnancy BMI were not statistically significantly correlated (r_s_ = 0.200, *p* = 0.385). Computing EPDS sum score as the fourth IV increased statistical significance of maternal pre-pregnancy BMI effect on neonate ReHo maps up to FWE corrected *p* < 0.001. Further, the spatial distribution of statistically significant results revealed altered right SFG ReHo values in addition to the original left SFG effect as two separate clusters. The cluster size for left SFG and right SFG were 487 and 645 voxels, respectively, at *p* < 0.001 level. The observed additive effects of included two IVs (EPDS sum score, gestational weight) likely stem from collinearity or from inclusion of too many IVs for a model with relatively small sample size.

### SCA

Group mean SCA revealed widespread functional connectivity (at *p* < 0.05 level, *p* < 0.001 FWE corrected) between left SFG seed and frontal regions and anterior cingulate gyrus (Supplementary materials, Fig. 7). No statistically significant effects were observed in the two-sample T-test when probing for group differences between neonates from mothers with pre-pregnancy BMI of ≥ 25.0 kg/m^2^ and controls at *p* < 0.05 level. Multiple regression analysis (Fig. 2) however revealed distinct positive and negative associations between functional connectivity (FC) and maternal pre-pregnancy BMI (at *p* < 0.05 level, *p* < 0.001 FWE corrected). Positive associations were observed between maternal pre-pregnancy BMI and left SFG FC to left lateral frontal gyrus, left lateral prefrontal cortex, left temporal pole and anterior temporal cortex, bilateral amygdala and left ventral striatum. Negative associations, in turn, were observed in prefrontal (including lateral, dorsal and medial portions), frontal (superior, lateral), temporal (anterior, left inferior), insular and basal ganglia.

## Discussion

In this study we explored whether maternal pre-pregnancy BMI affects neonate brain local and distal functional connectivity. We found that maternal pre-pregnancy BMI and neonate ReHo values were positively associated (FWE corrected *p* < 0.005, cluster size of 869 voxels) within the left SFG, suggesting that higher maternal BMI during pre-pregnancy or early pregnancy influences neonatal local brain connectivity. As a follow-up, we performed a SC analysis, using the left SFG as a seed region. SCA yielded distinct patterns of increased and decreased connectivity related to maternal pre-pregnancy BMI, suggestive of alterations in functional connectivity following overweight/obese pregnancy.

In neonates soon after birth, high ReHo values are encountered symmetrically in primary somatosensory and visual networks (mean ReHo map shown in Supplementary materials, Fig. 1 and^50^). Notably, previous developmental fMRI connectivity studies have estimated that these networks achieve adult-like network topology and function earlier than e.g. frontoparietal, executive control and default-mode networks^19,21,22^. In line with this idea, prior modelling studies have suggested an inverse relationship between distal connectivity and ReHo regarding a given voxel^46^, suggesting that, as functional segregation of networks ensues, ReHo values decrease. In this framework, our observation that ReHo in the left SFG was higher in neonates born to mothers with higher BMIs was suggestive of amplified local, and conversely, decreased distal connectivity in this region. This idea gained support also from our SC analysis, which revealed decreased distal and bilateral FC when maternal pre-pregnancy BMI was higher (Fig. 2). More explicitly, higher maternal pre-pregnancy BMI was associated with left-lateralized FC between left SFG and prefrontal and temporal regions. Positive associations were also observed bilaterally in the amygdalae and the ventral striatum with slight emphasis on the left side. Conversely, there was a negative association between maternal pre-pregnancy BMI and neonate FC regarding the left SFG and multiple bilateral regions within prefrontal cortices, temporal, thalamic and other basal ganglia regions as well as the insular cortices. These results are in line with prior work, which have demonstrated alterations related to maternal BMI in functional networks encompassing prefrontal, limbic and insular regions with emphasis on the left hemisphere^28,39^.

The left SFG has been identified as a key hub in the left frontoparietal network (FPN), which holds a central role in executive control, working memory and fluid intelligence in adults^51^. Furthermore, SFGs have been recognized as crucial areas for global networks in terms of network centrality in adults^52^ and identified as a possible connector hub between executive control network and default-mode-network^53^. However, in their immature state, brain networks in neonates likely have divergent functions as compared to corresponding networks in older infants and adults, complicating network-related change interpretation and comparison between populations of different age. For the left FPN, increases in within-network and inter-network connectivity between lateral visual, auditory/language and right FP networks, with simultaneous decreases in inter-network connectivity between medial visual and salience networks take place during the first year of life^21^. In light of previous studies into RSN development and ReHo interpretation, the observed positive association between maternal pre-pregnancy BMI and neonate left SFG ReHo values in this study may suggest accelerated within-network development. Here, we found left SFG FC patterns to become spatially decreased and lateralized to the left hemisphere in neonates born from obese/overweight pregnancies, suggesting modifications to the developing RSN distal connectivity. We observed a negative association for maternal pre-pregnancy BMI and FC between left SFG and central nodes that belong to the salience network, such as insular and anterior cingulate cortices. In reference to above discussed SFGs’ possible role as connector hubs, these findings may further indicate altered inter-network connectivity. Differences in salience network connectivity have been previously associated with impairment of shifting between task positive and task negative functional networks^54^. These inter-network divergences might underlie some of the observed cognitive performance differences seen in older children born from obese and overweight pregnancies^2,3^. Interestingly, the left SFG has been demonstrated to functionally couple with the anterior node of the default mode network (the ventromedial PFC)^55^ in adults. The reduced FC between left SFG and adjacent prefrontal regions after elevated maternal pre-pregnancy BMI may suggest disintegration of the DMN as an intra-network phenomenon, or conversely, disruption of connectivity between the left SFG (as a connector hub) and the DMN as an inter-network phenomenon involving FPN.

Prior investigations into maternal obesity and overweight during pregnancy related human infant neurodevelopment have revealed widespread functional connectivity and white matter tract alterations in the neonate brain^25–28^. Similarly, a recent study found that higher pre-pregnancy maternal BMI during gestation associated with variations in functional connectivity in fetal prefrontal, frontal and insular brain regions^39^. These results suggest that at least some group differences observed in obese/overweight and normal-weight populations could begin during the gestational period and may be attributed to metabolic, humoral and inflammatory processes in obese mothers. Indeed, obesity/overweight related changes in brain network organization have been well documented in adult populations with alterations emphasizing four distinct domains concerned with feeding behavior: sensory cue processing^56^, reward processing^57^, cognitive^56^ and motor control^58^. A recent seed-based connectivity study hypothesized that these network abnormalities could be conveyed through genetic or environmental effects and observed similar functional connectivity differences in neonates exposed to maternal obesity during gestation^28^. Similarly, we found a positive association between neonate FC and maternal pre-pregnancy BMI. This association localized to ventral striatum, amygdalae and left temporal and prefrontal regions, areas implicated in reward processing^56^. It remains important to address, whether these associations persist later during child development.

To the best of our knowledge, no structural MRI studies have been performed on neonates born from pregnancies with maternal obesity, but studies focusing on older obese/overweight children have found grey matter abnormalities within the frontal, prefrontal and limbic areas^59^. Moreover, the observed GM reduction were partly associated with impaired executive function^59^. These abnormalities largely spatially overlap with functional changes seen in neonates born from pregnancies with maternal obesity/overweight and likely precede structural abnormalities seen in older children and may begin as early as gestation.

Despite the observed widespread connectivity differences between neonates born from normal-weight and pregnancies with maternal obesity, it is unclear whether these changes are driven by systemic effects of insults or caused by localized impairment of key regions, e.g. connector hubs, followed by plasticity induced changes within plural functional networks, causing global differences in connectivity. It is likely that the impact of detrimental factors is not anatomically uniform, and that the most vulnerable regions are presumably crucial for networks that take years to reach maturity and obtain coherent function^16^.

## Limitations

We acknowledge that a larger sample size would have increased statistical power and possibly revealed more subtle local connectivity variations as well as allowed studying e.g. sex-specific associations. Similarly, due to the small sample size, we were unable to perform statistically reliable group difference tests for normal versus elevated BMI exposed subjects. Further, while BMI is a sound and frequently used indicator for obesity and overweight, it does not take into account the variability in body composition, e.g. fat and muscle ratios. This study unfortunately lacks background information on the types of maternal food intake, which is likely a contributing factor in obesity induced effects. Finally, no data was available for maternal BMI variability during the course of pregnancy and such data would be valuable in future studies (ideally coupled to other metabolic biomarkers).

## Conclusions

In this study, we showed that maternal pre-pregnancy BMI is positively associated with ReHo values within the neonate left SFG, suggesting an increase in local functional connectivity and amplified within-network connectivity. The increased ReHo in the left SFG is associated with decreased distal connectivity in neonates with exposure to higher maternal pre-pregnancy BMI. The reduced distal connectivity localizes to insular, basal ganglia and prefrontal regions. In addition, increased maternal pre-pregnancy BMI associates with increased left SFG connectivity between bilateral amygdalae and ventral striatum. These alterations in functional connectivity focus on regions pertinent for social and feeding behavior, as well as cognitive function. Our findings provide further evidence for maternal BMI influenced changes in functional brain development seen in neonates born from obese/overweight pregnancies. The observed alterations in functional connectivity within the left SFG are unlikely to be independently detrimental, and later outcome measures are needed in future studies.

## Data availability statement

The Finnish law and ethical permissions do not allow the sharing of the data used in this study.

## Supplementary Information

Supplementary Information.
