# Supplementary material for: Maternal pre-pregnancy BMI associates with neonate local and distal functional connectivity of the left superior frontal gyrus
Source: Sci Rep. 2021 Sep 28;11:19182. doi: 10.1038/s41598-021-98574-9 (PMC8478954; doi:10.1038/s41598-021-98574-9)
Supplement: Supplementary file 1 — Supplementary Information. [file 41598_2021_98574_MOESM1_ESM.docx]

**Supplementary material to**Maternal pre-pregnancy BMI associates with neonate local and distal functional connectivity of the left superior frontal gyrus

Olli Rajasilta^1^ MD, Suvi Häkkinen^1,11^ PhD, Malin Björnsdotter^9,10^ PhD, Noora M. Scheinin^1,2^ MD, PhD, Satu J. Lehtola^1^ MD, Jani Saunavaara^4^ PhD, Riitta Parkkola^5^ MD PhD, Tuire Lähdesmäki^7^ MD PhD, Linnea Karlsson^1,6^ MD PhD, Hasse Karlsson^1,2^ MD PhD, Jetro J. Tuulari^1,2,3,8^ MD, PhD.

1. FinnBrain Birth Cohort Study, Turku Brain and Mind Center, Institute of Clinical Medicine, University of Turku, Turku, Finland
2. Department of Psychiatry, University of Turku and Turku University Hospital, Turku, Finland
3. Department of Psychiatry, University of Oxford, UK (Sigrid Juselius Fellowship).
4. Department of Medical Physics, Turku University Hospital, Turku, Finland
5. Department of Radiology, University of Turku and Turku University Hospital, Turku, Finland
6. Department of Child Psychiatry, University of Turku and Turku University Hospital, Turku, Finland
7. Department of Pediatric Neurology, Turku University Hospital and University of Turku, Finland
8. Turku Collegium for Science and Medicine, University of Turku, Turku, Finland
9. The Sahlgrenska University Hospital, Gothenburg, Sweden
10. Department of Clinical Neuroscience, Karolinska Institutet, Stockholm, Sweden
11. Department of Neurology, University of California, San Francisco, San Francisco, CA, USA

Corresponding author
Olli Rajasilta | operaj@utu.fi
FinnBrain Birth Cohort Study, Turku Brain and Mind Center
Lemminkäisenkatu 2, 20520, Turku, Finland

The purpose of this supplementary material is to provide the reader with:

1. Mean ReHo maps of the neonate brain at 26.14 ± 6.28 days after birth.

2. Effects of additional independent variables on ReHo-BMI models (p < 0.005; p < 0.001):

a. Gestational weight

b. EPDS sum score

3. Cluster-level descriptions of the ReHo associations between pre-pregnancy maternal BMI and additional independent variables.

4. Statistical nonparametric mapping (SnPM) results of main ReHo model.

5. Mean SCA maps.

6. Statistical nonparametric mapping result of multiple regression SCA model.


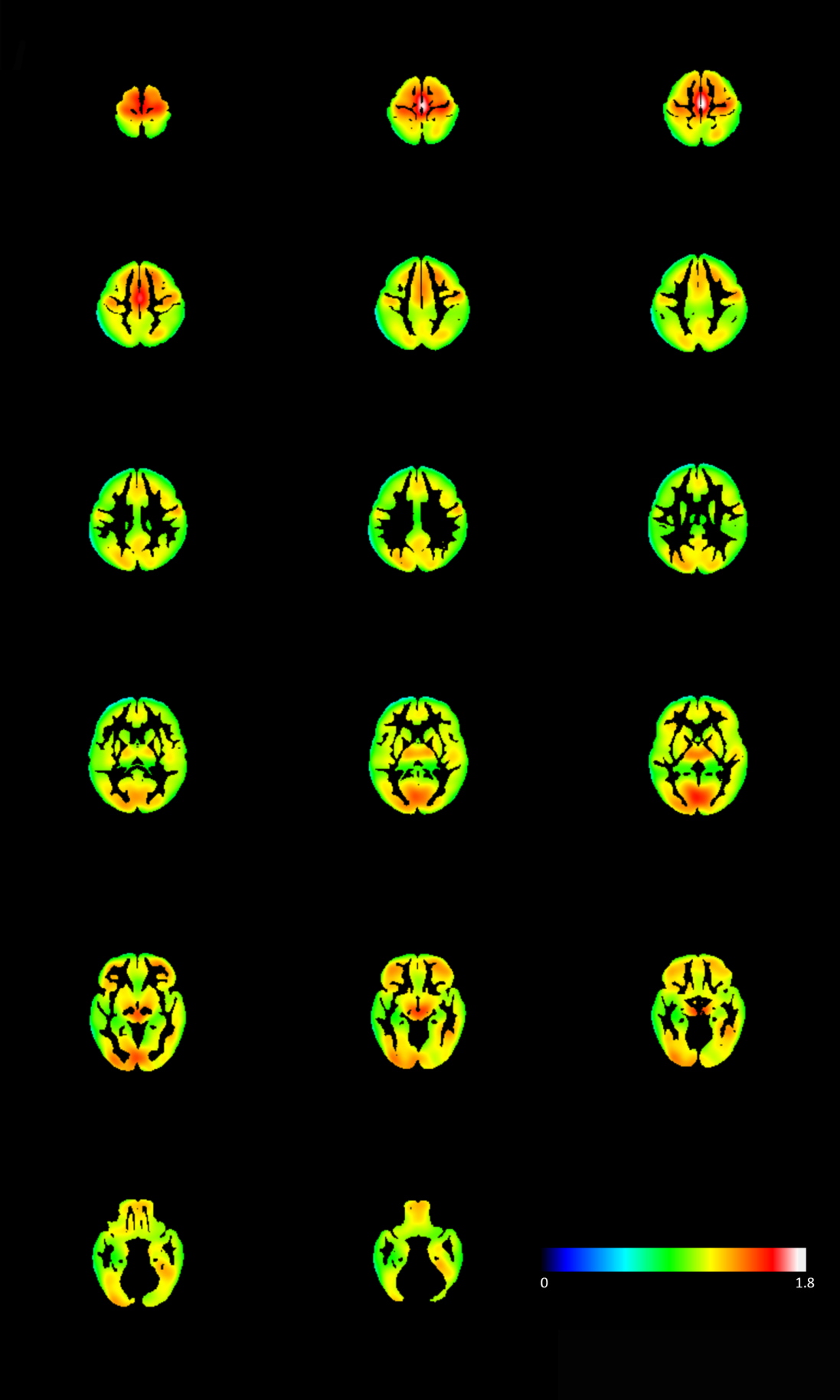
**1. Mean ReHo maps (N = 21) of the neonate brain at 26.14 ± 6.28 days after birth.**

**Supplementary materials, figure 1.** Mean ReHo maps (N = 21) of the neonate brain at 26.14 ± 6.28 days after birth. Color bar denotes mean ReHo values. High mean ReHo values are symmetrically concentrated on sensorimotor, visual and basal ganglia regions. Similar results were acquired in a recent comprehensive paper ^1^.

**2. Effects of additional independent variables on ReHo-BMI models.**


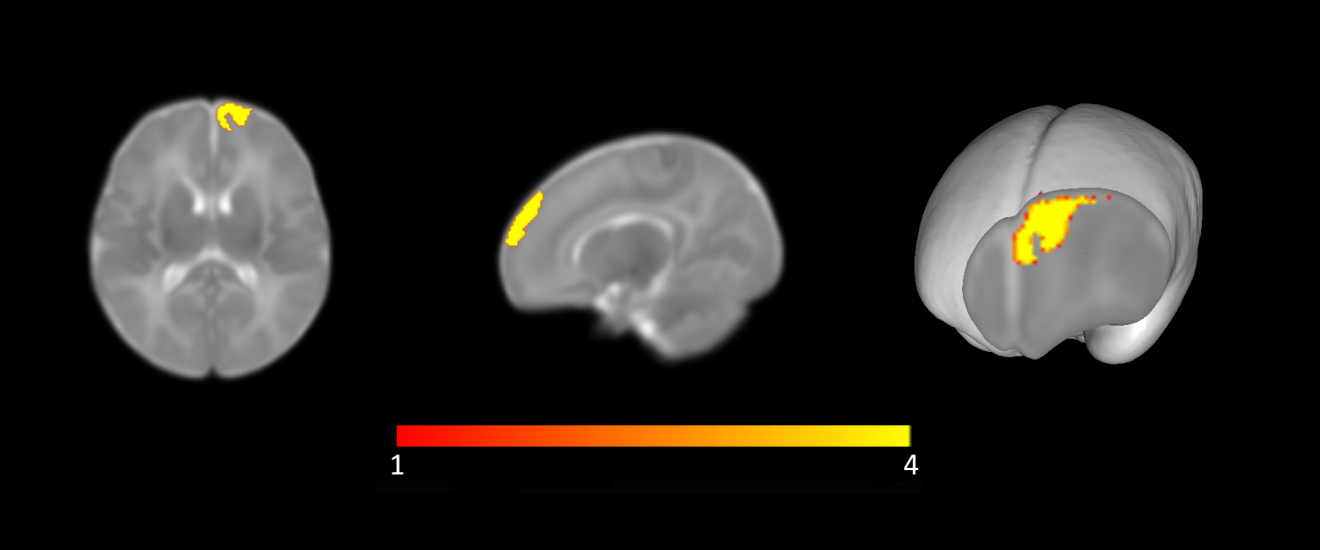
**a. Offspring birth weight**


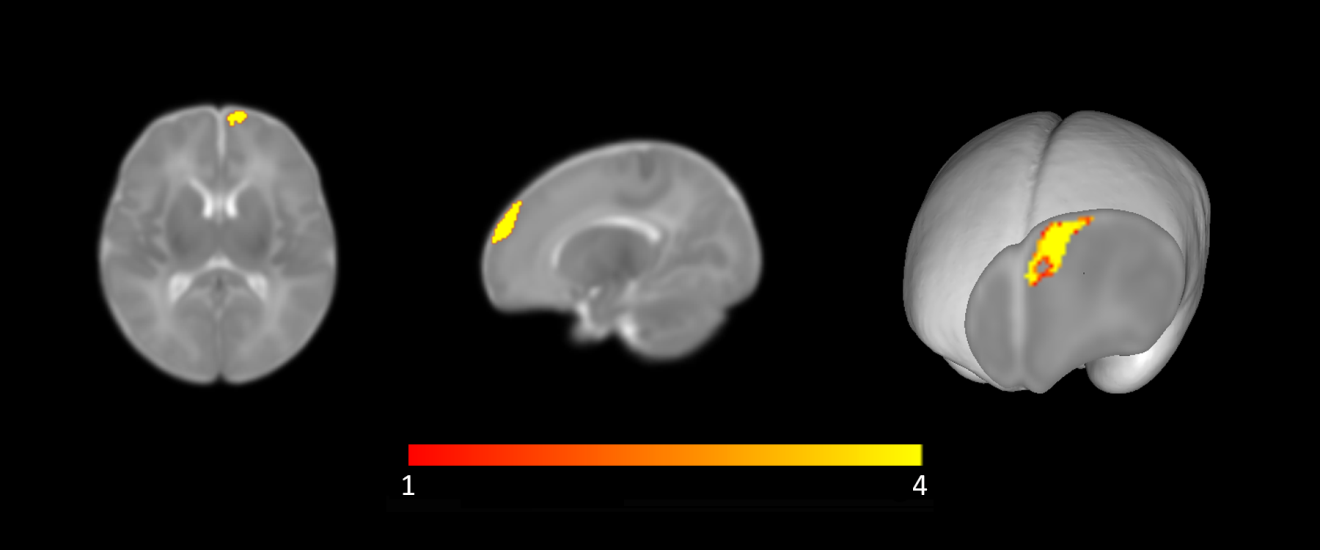
**Supplementary materials, figure 2.** Regions where ReHo significantly correlated with maternal pre-pregnancy BMI in a model with offspring birth weight as the third independent variable of no interest (p < 0.005 threshold; p < 0.001 FWE-corrected, kE 1437) highlighted left SFG within the naturally sleeping neonate (N = 21). Color bar represents t-scores. Images are displayed in radiological convention on the UNC neonate template in axial, sagittal and oblique slices. Cluster coordinates are displayed in Supplementary materials Table 1. Visualization was performed with Mango software version 4.0.1. Oblique slice was extracted from a generated surface model. Abbreviations: ReHo = regional homogeneity; SFG = superior frontal gyrus.

**Supplementary materials, figure 3.** Regions where ReHo significantly correlated with maternal pre-pregnancy BMI in a model with offspring birth weight as the third independent variable of no interest (p < 0.001 threshold; p < 0.001 FWE-corrected, kE 609) highlighted left SFG within the naturally sleeping neonate (N = 21). Color bar represents t-scores. Images are displayed in radiological convention on the UNC neonate template in axial, sagittal and oblique slices. Cluster coordinates are displayed in Supplementary materials, Table 1. Visualization was performed with Mango software version 4.0.1. Oblique slice was extracted from a generated surface model. Abbreviations: ReHo = regional homogeneity; SFG = superior frontal gyrus.


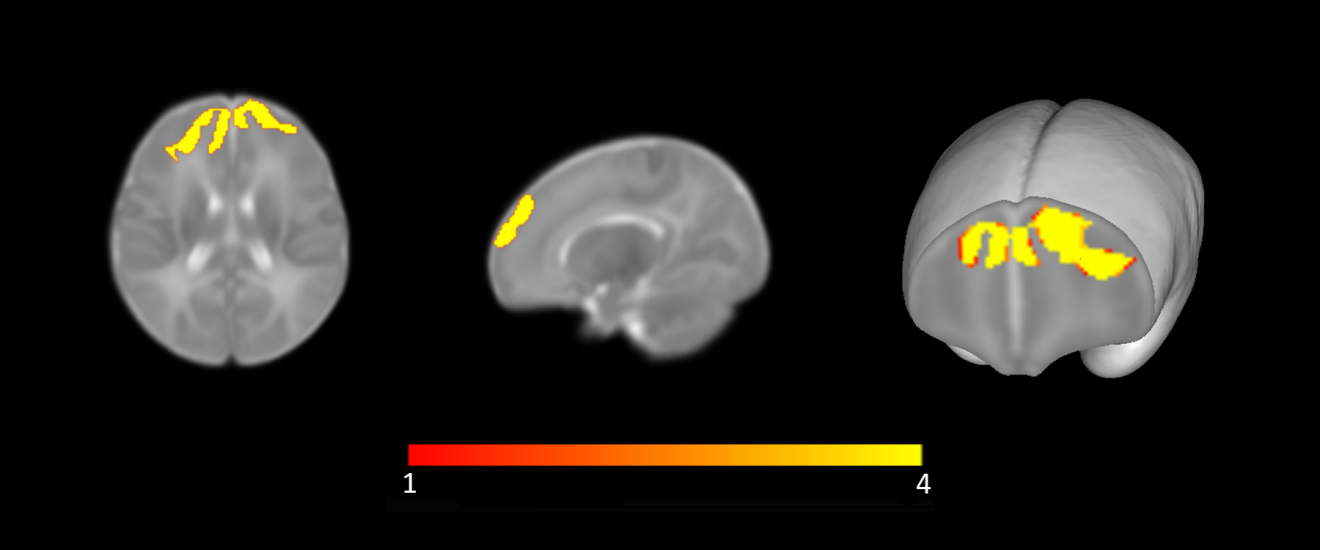
**b. EPDS sum score**


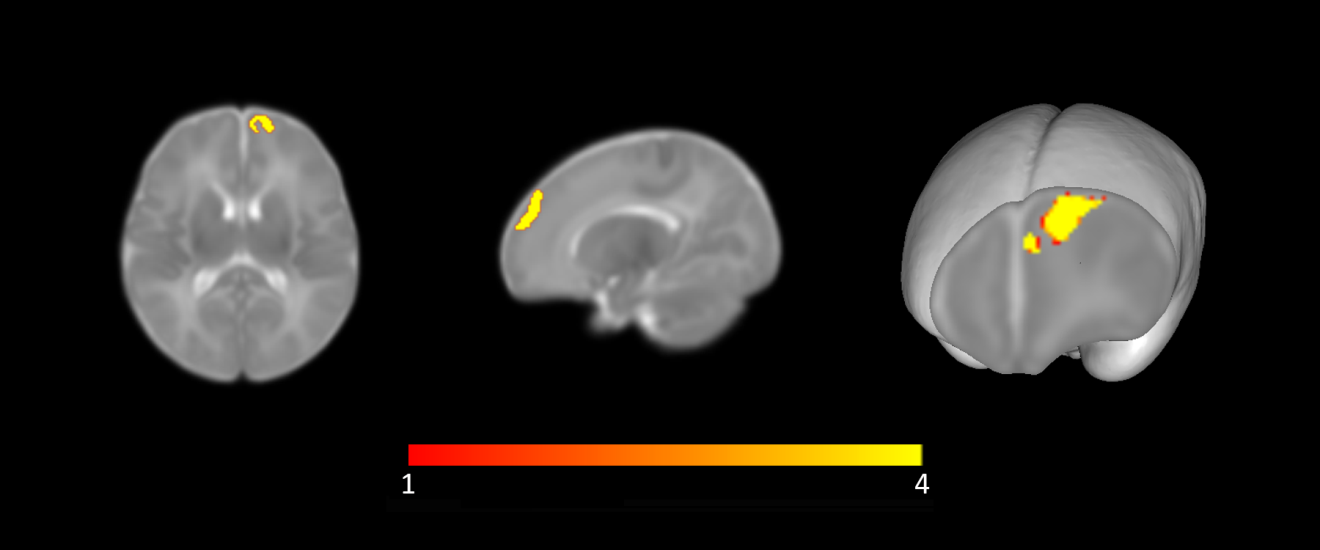
**Supplementary materials, figure 4.** Regions where ReHo significantly correlated with maternal pre-pregnancy BMI in a model with EPDS sum score as the third independent variable of no interest (p < 0.005 threshold; p < 0.001 FWE-corrected, kE 4493) highlighted right and left SFG within the naturally sleeping neonate (N = 21). Color bar represents t-scores. Images are displayed in radiological convention on the UNC neonate template in axial, sagittal and oblique slices. Cluster coordinates are displayed in Supplementary materials, Table 1. Visualization was performed with Mango software version 4.0.1. Oblique slice was extracted from a generated surface model. Abbreviations: ReHo = regional homogeneity; SFG = superior frontal gyrus.

**Supplementary materials, figure 5.** Regions where ReHo significantly correlated with maternal pre-pregnancy BMI in a model with EPDS sum score as the third independent variable of no interest (p < 0.001 threshold; p < 0.262/0.001 FWE-corrected, kE 487/645, respectively) highlighted left SFG in two separate clusters within the naturally sleeping neonate (N = 21). Color bar represents t-scores. Images are displayed in radiological convention on the UNC neonate template. Cluster coordinates are displayed in Supplementary materials, Table 1. Visualization was performed with Mango software version 4.0.1. Oblique slice was extracted from a generated surface model. Abbreviations: ReHo = regional homogeneity; SFG = superior frontal gyrus.

**3. Cluster-level descriptions of the ReHo associations between pre-pregnancy maternal BMI and additional independent variables.**

**Supplementary materials, table 1.** Original and additional model designs with additional independent variable corrected effects of pre-pregnancy maternal BMI on neonate ReHo maps. Abbreviations: EPDS = Edinburgh postnatal depression scale 10-point questionnaire score filled out by mothers at 24^th^ gestational week.

| **Original model (**independent variables of no interest and **explanatory variable)** | **p-threshold** | **p (FWE-correction)** | **Cluster size in voxels (kE)** | **Cluster coordinates** (UNC-neonate-template space) **(x, y, z)** |
| --- | --- | --- | --- | --- |
| - Gestation corrected age - Neonate sex - **Maternal pre-pregnancy BMI** | 0.005 | 0.002 | 869 | 8, 48, -3 11, 44, 7 |
| **Models** with additional independent variable of no interest and **explanatory** **variable** | | | | |
| - Gestation corrected age - Neonate sex - **Maternal pre-pregnancy BMI** - Apgar points at 1 minute | 0.005 | 0.003 | 812 | 9, 55, -7 8, 48, -3 11, 44, 7 |
| - Gestation corrected age - Neonate sex - **Maternal pre-pregnancy BMI** - Apgar points at 5 minutes | 0.005 | 0.001 | 988 | 11, 44, 7 5, 45, -1 7, 51, -6 |
| - Gestation corrected age - Neonate sex - **Maternal pre-pregnancy BMI** - Maternal age | 0.005 | 0.985 | 154 | 8, 53, -8 |
| - Gestation corrected age - Neonate sex - **Maternal pre-pregnancy BMI** - EPDS sum score | 0.005 | 0.000 | 4493 | -8, 47, -2 13, 44, 6 9, 47, -3 |
|  | 0.001 | 0.262  0.000 | 487  645 | -8, 47, -2  13, 44, 6 9, 47, -3 |
| - Gestation corrected age - Neonate sex - **Maternal pre-pregnancy BMI** - Gestational weight | 0.005 | 0.000 | 1437 | 11, 45, 7 6, 53, -7 21, 41, 7 |
|  | 0.001 | 0.000 | 609 | 11, 45, 7 6, 53, -7 |

**Supplementary materials, table 2.** The nonparametric Spearman rank correlation coefficients between all explanatory and independent variables. ** denotes significant correlation at the p < 0.01 level. Abbreviations: EPDS = Edinburgh postnatal depression scale 10-point questionnaire score filled out by mothers at 24^th^ gestational week.

| **Maternal pre-pregnancy BMI** |  | **Maternal pre-pregnancy BMI** | **Apgar points at 1 minute** | **Apgar points at 5 minutes** | **Maternal age** | **EPDS sum score** | **Gestational weight** |
| --- | --- | --- | --- | --- | --- | --- | --- |
|  | Correlation coefficient (r_s_) | 1.000 | 0.035 | 0.054 | 0.570** | -0.342 | 0.200 |
|  | Statistical significance (p) | - | 0.880 | 0.817 | 0.007 | 0.129 | 0.385 |
| **Apgar points at 1 minute** | Correlation coefficient (r_s_) | 0.035 | 1.000 | 0.672** | 0.064 | -0.127 | 0.241 |
|  | Statistical significance (p) | 0.880 | - | 0.001 | 0.782 | 0.584 | 0.292 |
| **Apgar points at 5 minutes** | Correlation coefficient (r_s_) | 0.054 | 0.672** | 1.000 | -0.182 | -0.026 | 0.146 |
|  | Statistical significance (p) | 0.817 | 0.001 | - | 0.430 | 0.910 | 0.527 |
| **Maternal age** | Correlation coefficient (r_s_) | 0.570 | 0.064 | -0.182 | 1.000 | -0.112 | -0.038 |
|  | Statistical significance (p) | 0.007 | 0.782 | 0.430 | - | 0.629 | 0.871 |
| **EPDS sum score** | Correlation coefficient (r_s_) | -0.342 | -0.127 | -0.026 | -0.112 | 1.000 | -0.675** |
|  | Statistical significance (p) | 0.129 | 0.584 | 0.910 | 0.629 | - | 0.001 |
| **Infant birth weight** | Correlation coefficient (r_s_) | 0.200 | 0.241 | 0.146 | -0.038 | -0.675** | 1.000 |
|  | Statistical significance (p) | 0.385 | 0.292 | 0.527 | 0.871 | 0.001 | - |

**4. Statistical nonparametric mapping results of the main model**

In this model, we applied statistical nonparametric mapping strategy for investigating the main model effect by implementing SnPM13 (<https://warwick.ac.uk/fac/sci/statistics/staff/academic-research/nichols/software/snpm>) software. This permutation-based analysis was performed to ensure the validity of our primary statistical parametric mapping findings. Otherwise identical model design took place as in the main model: Maternal pre-pregnancy BMI was set as the main explanatory variable and gestation corrected age and neonate sex were set as primary independent variables. Statistical threshold was set at p < 0.005 and corrected with FWE at cluster level. Images were inclusively masked with averaged UNC neonate template GM mask to limit the statistics to grey matter.


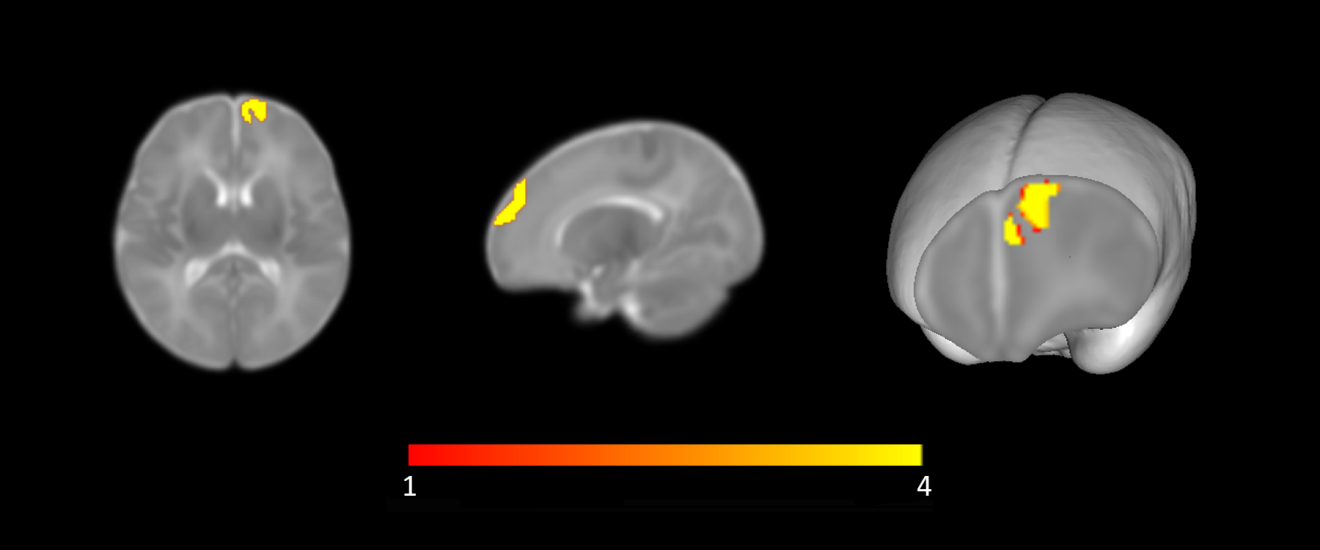
The SnPM approach resulted in a highly similar cluster pattern albeit with marginally smaller cluster size (kE = 791) as the main SPM model (kE = 869), owing to the inherent reduced statistical power of nonparametric testing. We regard the main SPM model as our primary result and the SnPM model to cement our main findings.
**Supplementary materials, figure 6.** Regions where ReHo significantly correlated with maternal pre-pregnancy BMI in a nonparametric model (p < 0.005 threshold; p < 0.004 FWE-corrected, kE 791) highlighted left SFG in three separate clusters within the naturally sleeping neonate (N = 21). Cluster coordinates are: 9, 47, -3; 11, 44, 7; 23, 47, -8 (in x, y, z planes, respectively, in UNC-neonate-template space) Color bar represents t-scores. Images are displayed in radiological convention on the UNC neonate template. Visualization was performed with Mango software version 4.0.1. Oblique slice was extracted from a generated surface model. Abbreviations: ReHo = regional homogeneity; SFG = superior frontal gyrus.


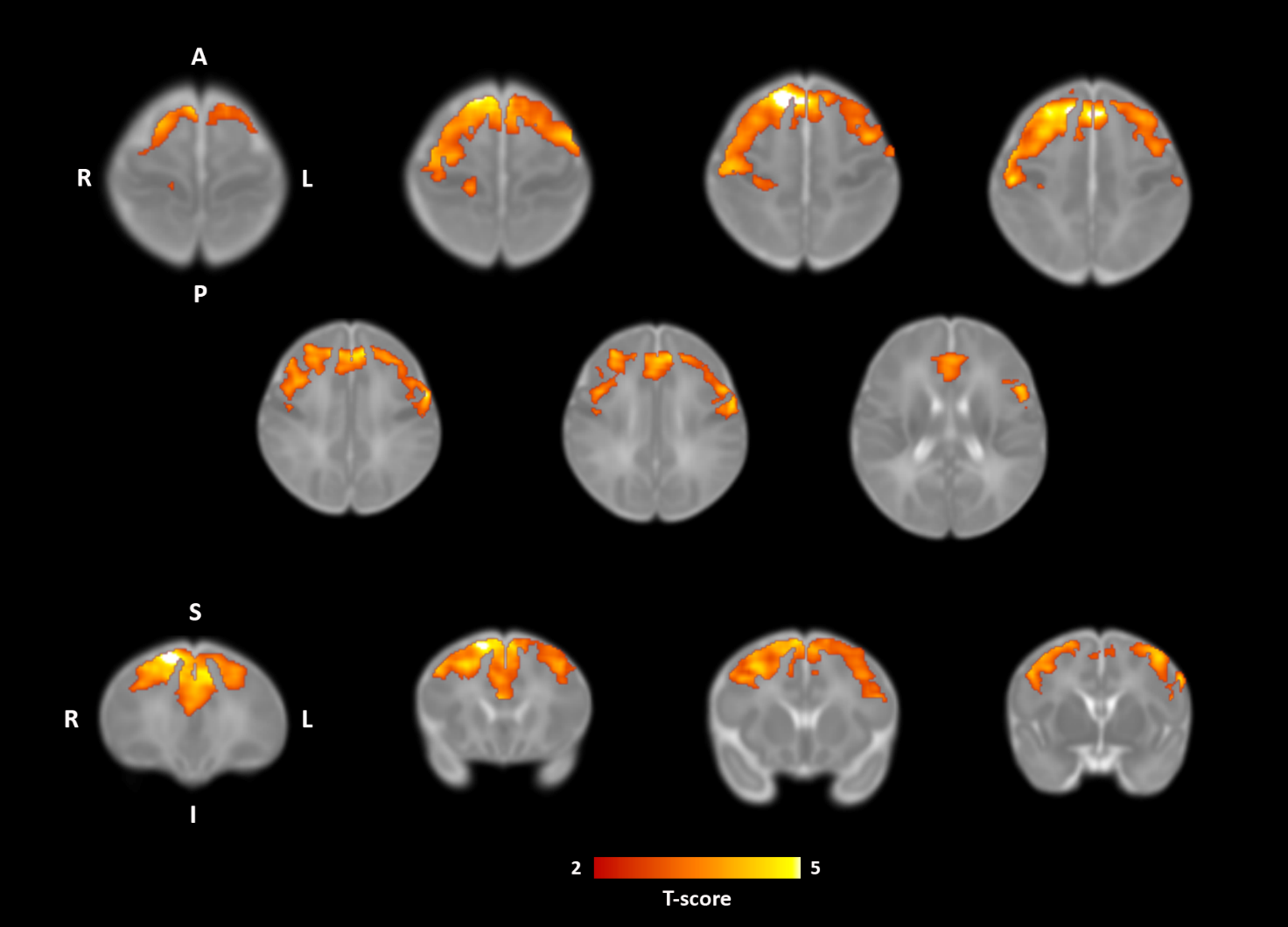
**5. Mean SCA maps.**

**Supplementary materials, figure 7.** Mean connectivity from seed ROI (left SFG) in the neonate brain (N = 21). Seed ROI location is illustrated in Figure 2. Color bar represents T-scores. Images are displayed in radiological convention on the UNC neonate template. Visualization was performed with Mango software version 4.0.1. Abbreviations: SFG = superior frontal gyrus; ROI = region-of-interest, A = Anterior, P = Posterior, R = Right, L = Left.


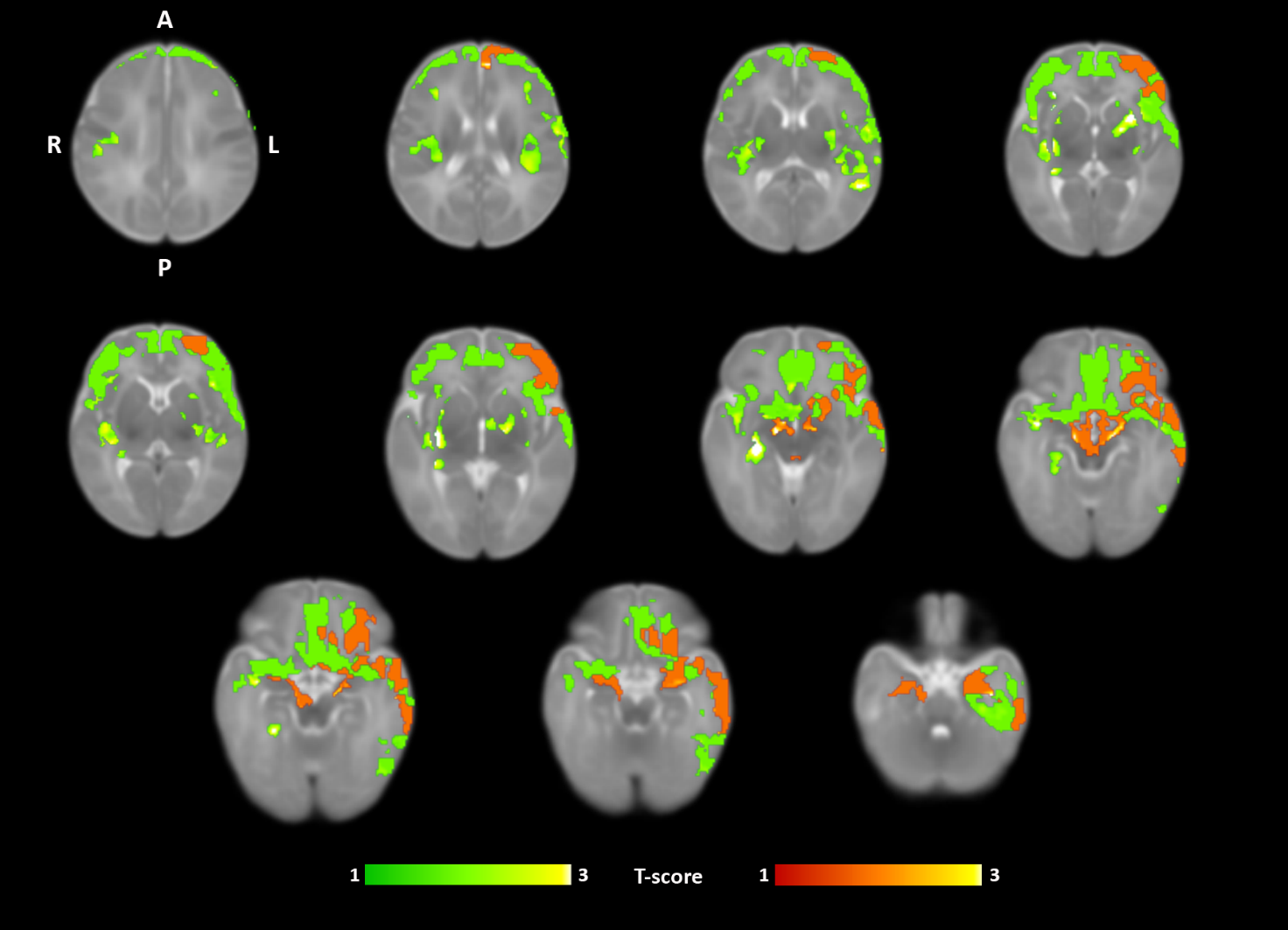
**6. Statistical nonparametric mapping result of multiple regression SCA model.**

**Supplementary materials, Figure 8.** Statistical non-parametric mapping results of the main SCA model. Regions where left SFG functional connectivity was positively (orange) and negatively (green) correlated with maternal pre-pregnancy BMI (p < 0.05 threshold; p < 0.001 FWE-corrected) in the sleeping neonate (N = 21). Color bars represent T-scores. Images are displayed in axial slices on the UNC neonate template. These nonparametric results showed striking overlap with those obtained with parametric models. Abbreviations: SFG = Superior frontal gyrus, A = Anterior, P = Posterior, R = Right, L = Left.

**References**

1. Huang, Z. *et al.* Exploring functional brain activity in neonates: A resting-state fMRI study. *Dev. Cogn. Neurosci.* **45**, (2020).
